# NLM-Chem, a new resource for chemical entity recognition in PubMed full text literature

**DOI:** 10.1038/s41597-021-00875-1

**Published:** 2021-03-25

**Authors:** Rezarta Islamaj, Robert Leaman, Sun Kim, Dongseop Kwon, Chih-Hsuan Wei, Donald C. Comeau, Yifan Peng, David Cissel, Cathleen Coss, Carol Fisher, Rob Guzman, Preeti Gokal Kochar, Stella Koppel, Dorothy Trinh, Keiko Sekiya, Janice Ward, Deborah Whitman, Susan Schmidt, Zhiyong Lu

**Affiliations:** National Library of Medicine, National Institutes of Health, Bethesda, MD 20894 USA

**Keywords:** Data mining, Cheminformatics, Statistical methods, Machine learning, Literature mining

## Abstract

Automatically identifying chemical and drug names in scientific publications advances information access for this important class of entities in a variety of biomedical disciplines by enabling improved retrieval and linkage to related concepts. While current methods for tagging chemical entities were developed for the article title and abstract, their performance in the full article text is substantially lower. However, the full text frequently contains more detailed chemical information, such as the properties of chemical compounds, their biological effects and interactions with diseases, genes and other chemicals. We therefore present the NLM-Chem corpus, a full-text resource to support the development and evaluation of automated chemical entity taggers. The NLM-Chem corpus consists of 150 full-text articles, doubly annotated by ten expert NLM indexers, with ~5000 unique chemical name annotations, mapped to ~2000 MeSH identifiers. We also describe a substantially improved chemical entity tagger, with automated annotations for all of PubMed and PMC freely accessible through the PubTator web-based interface and API. The NLM-Chem corpus is freely available.

## Abstract (summary)

**Table:** 

Measurement(s)	chemical entity • textual entity • Annotation
Technology Type(s)	expert manual annotation • digital curation • machine learning

Machine-accessible metadata file describing the reported data: 10.6084/m9.figshare.13486839

## Background & Summary

Chemical names are one of the most searched entity types in PubMed^1^, and chemical entities appear throughout the biomedical research literature, encompassing studies from various disciplines beyond chemistry, such as medicine, biology, and pharmacology, etc. Correctly identifying chemical names has significant impact beyond literature search by helping scientists better understand their structure, usage and interactions with other molecular entities, for example in drug development research^2^.

Following Krallinger *et al*.^3^, identifying chemical names is especially challenging because:

- The variety of language expressions that authors from different disciplines use to refer to chemicals are often at odds with the chemical naming rules defined by standardization bodies.
- Chemical name variants can include an innumerable mix of typographical variants, alternating uses of hyphens, brackets, spacing, and word order. (For example: *5*,6*-Epoxy-8,11,14-eicosatrienoic acid*, 5*,6-EET, 5(6*)*epoxyeicosatrienoic acid, 5(6)-oxido-8,11,14-eicosatrienoic acid, 5(6)-oxidoeicosatrienoic acid, 5,6-epoxyeicosatrienoic acid*)
- Chemical entities have many synonyms. For example, all the following names refer to the same entity: *triphenyl phosphate, TPP, TPHP, OP(OC*_*6*_*H*_*5*_*)*_3_*, phosphoric acid, triphenyl ester, CAS: 115-86-6, ((PhO)3PO))*.
- Chemical mentions can refer to mixtures of chemicals. (N-phenyltrifluromethylcarbohydrazonoyl benzenesulfonate)
- Chemical names are often ambiguous, especially when abbreviated. For example, the abbreviation “MTT” can be mapped to more than 800 different strings in PubMed, including *3-(4,5-dimethylthiazol-2-yl)-2,5-diphenyltetrazolium bromide, methyl thiazolyl tetrazolium, mean transit time, malignant triton tumour*, and *myoblast transfer therapy*.
- Novel chemical names refer to chemical compounds that are newly discovered, or not catalogued before.
- Chemical entities’ complexity makes it difficult to identify exact boundaries for entity mentions and word tokens. For example: *dipotassium 2-alkylbenzotriazolyl bis(trifluoroborate)s*, *4,7-dibromo-2-octyl-2,1,3-benzotriazole, etc*.

These difficulties are often compounded in articles’ full text, compared with the title and abstract^4^, causing a substantial performance reduction in automated chemical named entity recognition (NER) systems trained using only titles and abstracts. However, the full text frequently contains more detailed chemical information, such as the properties of chemical compounds, their biological effects and interactions with diseases, genes and other chemicals. Developing a chemical entity recognition system that accurately addresses these challenges requires a manually-annotated corpus of chemical entities^3,5^, with sufficient examples in full-text articles for system training^6–8^ and an accurate evaluation of their performance.

The creation and maintenance of chemical terminologies^2^, and chemical corpora^9^, are a major concern in the biomedical community^10^. Recent efforts have developed several valuable chemical corpora for text mining, such as the CHEMDNER corpus^3^, the BC5CDR corpus^11^, and the CRAFT corpus^6^. The CHEMDNER corpus established a comparative evaluation for chemical named entity identification and a body of annotation guidelines defining what constituted a chemical compound mention in the biomedical text literature. The BC5CDR corpus assessed the identification of chemical-disease relations in biomedical text, but it contained annotations for both chemical mentions and normalized concept identifiers, using Medical Subject Headings (MeSH) as the controlled vocabulary. MeSH concepts are useful as chemical identifiers because they can be mapped to other vocabularies via UMLS and they are provided for each article in the MEDLINE metadata. The CRAFT corpus is a collection of full-text PMC articles with comprehensive annotations for many biomedical entities, including chemical annotations normalized to ChEBI identifiers. These corpora have served as valuable resources for the text mining community in the recent years^5,12–16^, however a corpus of annotated full-text articles with a focus on chemicals does not currently exist.

Our work makes these significant contributions:

First, we present a new high-quality, manually annotated corpus on chemicals for biomedical literature. The NLM-Chem corpus contains 150 full-text journal articles selected both to be rich in chemical mentions and for articles where human annotation was expected to be most valuable. These characteristics make this corpus invaluable for improving text mining tools for accurate chemical entity identification.

Second, we build a new end-to-end chemical NER system and demonstrate improved performance in full-text articles compared to TaggerOne^17^. This system uses bluebert^18^ for named entity recognition, a deep-learning based contextualized language model, trained on biomedical and clinical text. It also introduces the multi-terminology candidate resolution (MTCR) normalization architecture, which employs multiple string-matching methods, multiple chemical terminologies and ambiguity resolution to improve chemical entity identification.

Finally, our new resource is publicly available at Dryad^19^ and https://www.ncbi.nlm.nih.gov/research/bionlp/Data and the chemical entity recognition results are available via API https://www.ncbi.nlm.nih.gov/research/bionlp/APIs/ and/or Pubtator: https://www.ncbi.nlm.nih.gov/research/pubtator/.

## Methods

### The NLM chemical corpus

The NLM Chemical (NLM-Chem) corpus was created iteratively, beginning with defining the annotation goal. We then reviewed the procedures used for previous annotation projects for both chemicals and other named entities.

Following our previous work on biomedical article annotation^7,20,21^, and especially^3^ on chemical entity annotation, we carefully considered several critical aspects that could influence the corpus quality. These were:

- Selection and sampling of full-text articles to annotate
- Annotation guidelines
- Human annotator expertise and training
- Annotation tools and interface
- Annotation process and inter-annotator consistency
- Corpus format and availability

### Document selection

The NLM-Chem corpus document set had to be representative of biomedical literature publications that contain chemical mentions. In addition, we decided to have this document set be complementary to other Chemical entity recognition corpora such as CHEMDNER or BC5CDR; therefore, it was decided that the selection would target articles for which human annotation was most valuable. Fundamentally, this set would be instrumental in training Chemical NER algorithms to produce high-quality results in full-text publications and article abstracts.

We identified a set of constraints in order to select candidate articles for human annotation:

1. The data should be rich in chemical entities that current NER tools have trouble identifying
2. The data should have no restrictions on sharing and distribution
3. The data could potentially be useful for other downstream tasks related to biomedical entity text mining

In order to optimize for the constraints listed above, we designed the following procedure:

- We began with all articles from the Open Access Dataset with no licensing restrictions (CC-BY or CC0 license).
- We next ran several filtering steps:
  - We filtered articles that did not have an abstract and at least one reference article.
  - We filtered articles that were retracted, a correction, or an editorial.
  - We filtered abnormally long articles, as measured by the number of BioC passages (roughly equivalent to text paragraphs). We set the limit at 152 passages, three standard deviations (3sigma) above the mean (71.0 passages).
  - We filtered articles for which our tools did not predict any chemical entities.
- We created a sample of articles for detailed analysis by separating the articles remaining after filtering into two sets; one set was published in journals whose title contains either “chem” or “molec” (case insensitive), and the other set was published in any other journal. We then randomly selected 10 K from each set, for a total of 20 K articles, to ensure that the sample retained high diversity while also enriching the sample with articles likely to contain many chemical entity mentions.
- We ran our suite of biomedical named entity recognition tools (PubTator^12^) and gave a higher weight to articles containing at least one non-chemical biomedical entity in the abstract (either disease, gene, mutation or species).
- We ran four different chemical entity recognition models and gave a higher weight to articles with a high level of disagreement between the different chemical recognition tools. The chemical named entity recognition models were:

1. The chemical annotations provided by PubTator, created using tmChem^22^ model 2. This system combines a machine-learning model for named entity recognition with a dictionary approach for identifying the recognized concepts.
2. The predictions from tmChem model 1 trained using a combination of the CHEMDNER corpus and the chemicals from the BC5CDR corpus. This system also combines a machine-learning model for named entity recognition with a dictionary approach for identifying the recognized concepts.
3. The predictions from tmChem model 1 but trained instead using a combination of the CHEMDNER corpus and CEMP corpus^5^.
4. TaggerOne 0.2.3, a system that uses machine learning to model both named entity recognition and normalization simultaneously. This model was trained on a combination of the chemicals present in the BC5CDR corpus and the CHEMDNER corpus. Since TaggerOne requires concept-level annotations that the CHEMDNER corpus does not provide, TaggerOne was updated with a mixed training mode. This mode trains the model using the concept annotation if provided by the source corpus and the concept annotation provided by the normalization model if not (similar to expectation maximization).

- In rounds 2 and 3 we gave a higher weight to articles dissimilar from those already selected. This was calculated by first converting each of the articles to a TF-IDF vector, using token counts from all of PubMed and PMC, then determining the cosine similarity between the centroid of the articles selected in previous rounds and the articles being considered. Articles with lower similarities were weighted more heavily. The primary effect of this procedure was to select articles for manual annotation containing vocabulary not already present in the corpus, though it also tended to prefer articles containing uncommon words.
- All weights were combined into a scoring method, including a random weight to increase diversity. The PMC Open Access articles described above were ranked, and the top ones were selected for each batch and manually reviewed to ensure topic diversity. The full text of each article was downloaded in BioC format using the NCBI BioC API for PubMed Central Open Access^23^. References and tables were removed manually but table captions were retained. Pre-annotations were created using TaggerOne 0.2.3 with the model trained on both the BC5CDR and CHEMDNER corpora, and the annotations made in previous batches of the NLM-Chem corpus. These articles were uploaded into the annotation tool and manually annotated as described in the next section.

Table 1 illustrates the difference in the average number of predicted entities for the selected 20 K PMC chemical-rich set of articles, and the articles ultimately selected for human annotation for the NLM-Chemical corpus. As noted, the articles selected for manual annotation are enriched in chemical entities.

**Table 1:** Difference in the number of PubTator predictions in a general set of PubMed articles, selected to be moderately enriched in chemical mentions, and the articles selected for annotation in the NLM-Chem corpus.

	Annotations per abstract in 20 K PubMed articles	Annotations per abstract in NLM-Chem corpus
Chemicals	3.98	14.58
Species	6.23	3.45
Gene	5.91	6.29
Disease	5.07	5.69
Mutation	0.06	0.37

### Annotation guidelines

Our goal for the NLM Chemical corpus is to be completely transparent in the annotation guidelines developed for its creation so that it can be extended, used, and compared and/or combined in a meaningful way with other chemical annotation corpora for the development and improvement of chemical name entity recognition tools. The complete NLM Chemical corpus annotation guidelines are publicly available with the corpus. Here we give a quick summary and encourage readers to refer to the supplementary file for details and examples.

Our guidelines specify which text elements should or should not be annotated and how to assign the annotated mentions to their corresponding MeSH identifiers. The primary considerations in the annotation guidelines are: (a) what should be labelled as a chemical, (b) how to place the mention boundaries for those labels, and (c) how to associate those mentions with an entity within one of the chemical trees of MeSH.

Creating high-quality guidelines that fit the annotation task required a multi-step iterative process, starting from an initial draft that was revised until clear and refined guidelines were obtained. We found that determining which text spans constitute chemical mentions required significant knowledge of chemistry, combined with careful consideration of the texts’ meaning and consulting additional knowledge sources as needed. Our chemical annotation guidelines were prepared by 10 professional MeSH indexers with degrees in Chemistry, Biochemistry, Biological Sciences, and Molecular Biology and an average of 20 years of experience in indexing PubMed literature with Medical Subject Heading indexing terms.

First, it was decided that very general chemical concepts (such as atom(s), moiety (moieties)) and terms that cannot be associated directly with a chemical structure such as molecule(s), drug(s), and polymer(s) were excluded from the annotation process. In addition, macromolecular biochemicals, namely, proteins (including enzymes), lipids, nucleic acids (DNA, RNA) were excluded from the annotation. Embedded chemical concepts in other biomedical entities such as “sodium channel gene,” where the chemical concept “sodium” is embedded in a phrase indicating a different type of biochemical entity “gene,” were tagged as OTHER. Each rule defined in the guidelines was represented by at least one illustrative example to simplify comprehension and application.

### Pre-annotation guideline discussions

At the start of the project, before the full-text articles of the NLM Chemical corpus were selected, the annotators had several meetings and jointly worked on the full annotation of one randomly selected full-text article. These meetings introduced the guidelines and posed questions to improve the guidelines. The first meeting also introduced the annotation tool, and each consecutive meeting also highlighted the necessity of introducing certain functions to ease the annotation process. After completing annotation of the NLM-Chem corpus, as a result of these efforts, we developed a new and improved collaborative annotation tool, TeamTat^24^.

### Pilot annotation guidelines development

For ten consecutive weeks, the annotators fully annotated one full-text article each week and discussed their work in the weekly meeting. These discussions further developed the annotation rules and provided examples to ease understanding and application. The annotation guidelines were reformulated when ambiguities or inconsistencies were detected. During this stage, all annotators worked on all full-text documents. All annotations were then compared in the weekly meeting, and the differences were discussed. This experiment allowed us to estimate the time required for annotation and to refine the guidelines according to annotator feedback to improve comprehension and repeatability. This also provided time for the annotators to learn how to use the annotation interface and for the developers to adjust the interface to better meet the needs of annotating mentions according to the guidelines.

### Corpus annotation

**Fig. 1:**
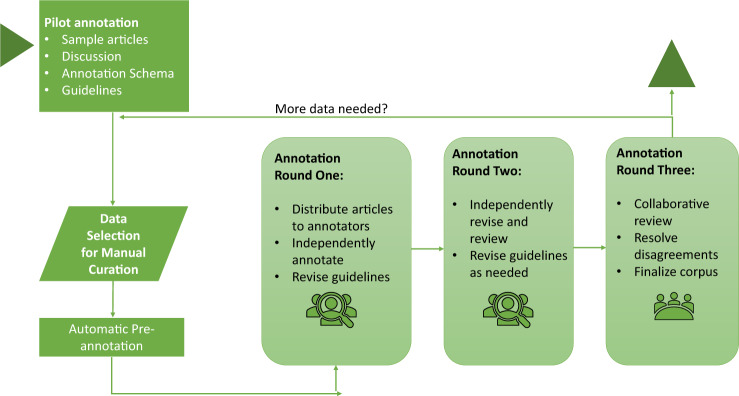
Annotation process.

The NLM-Chem corpus annotation consisted of three rounds as shown in Fig. 1.

All articles are doubly annotated. The first round of annotations consisted of each annotator annotating their assigned articles independently, without knowing the identities of their partners. The technical team reviewed these annotations to identify differences and discrepancies. The two sets of annotations for each document were then merged, with agreements and disagreements were marked in different colors. The second round of annotations consisted of each annotator independently working on a copy of the merged documents, without revealing their annotation partners’ identities. Each annotator reviewed their own decisions, considered their partners’ decisions, and edited the documents until they were satisfied. After the completion of this round, the set of annotations was again reviewed by the technical team to identify differences and discrepancies. All annotations for each document were again merged, with agreements and disagreements marked in different colors, and the documents made available to the respective annotators’ accounts. In the third and final round of annotations, the annotation partners for each document were revealed. The partners collaboratively reviewed and discussed any remaining annotation differences until complete consensus was reached and finalized the shared document.

Several measures were taken in the design of the annotation process to ensure that annotators were distributed an equitable load, and to ensure a group consensus and general corpus quality without bias. The NLM-Chem corpus was annotated in three batches. Batches 1 and 2 contained 45 full-text articles, and the third batch contained 60 full-text articles. In batches 1 and 2 every annotator annotated nine articles and each annotator was paired with every other annotator exactly once. Batch 3 followed the same procedure using sixty full-text articles, so that each annotator was paired with every other annotator once or twice. The article assignment was also arranged so that the total number of tokens, number of paragraphs (i.e. article length), and the number of assigned pre-annotations were approximately equal for each annotator. Lastly, following the last annotation round, each annotator was responsible for the final review of a given number of articles. Again, to balance the workload, the articles were assigned so that the total number of disagreements was distributed roughly equally.

### Corpus annotation experts and annotation interface

The NLM-Chem corpus was developed by highly qualified NLM indexers. They have extensive experience (ranging from 10 to 40 years) in indexing PubMed articles using MeSH terms. They are thus very familiar with the biomedical literature from a variety of fields including clinical medicine, chemistry, biochemistry, molecular biology, genetics, cell biology and other life sciences fields. Most have advanced degrees in one of the biological sciences or chemistry. This group was uniquely qualified to perform this task, because they have the most suitable academic backgrounds and experience with biomedical literature and annotation of this literature with Medical Subject Heading Terms. The only training the annotators needed was how to use the annotation interface to mark up the chemical mentions in the text at the character level and highlight all occurrences.

The TeamTat web-based annotation tool was selected for this project because it supports team annotation and is fully integrated with PubMed and PMC. TeamTat has an intuitive interface, it does not require local installation, it supports pre-annotation, and it accurately captures Unicode character offsets. The annotation tool allows quick and easy browsing of the full-text articles, for easy markup and editing functions. The tool is also capable of linking to the MeSH browser to select correct identifiers. The tool is equipped with links to the PubMed and PubMed Central versions of the articles in consideration, as well as a tabular view of the annotated mentions.

All articles were pre-annotated using the NCBI text mining suite TaggerOne tool. Prior to each new annotation batch, TaggerOne was retrained using all the chemicals in the BC5CDR, CHEMDNER, and all the available data in the NLM Chem corpus. This version of the tool was used to provide automated predictions for the new batch of documents, which then were uploaded to the TeamTat system, and distributed to the annotator accounts. Annotators were explicitly instructed to ignore the pre-annotations if inaccurate or if in doubt.

Annotators were encouraged to crosscheck information to ensure that the annotations reflected the meaning of the text and were consistent with the guidelines. Annotators were free to reference other sources as needed, including chemical databases (ChEBI, DrugBank, PubChem, etc.) or to search for information online.

TeamTat is an annotation interface developed and enriched with features and functionalities that allow for a better annotation experience such as: automatic search, automatic annotation of all occurrences of a given string throughout the text, easy access to MeSH browser for cross checking the normalization, an intelligent bookmark function that remembers the last paragraph the annotators were working on, and visual clues for annotators to mark individual annotations that have been checked, reviewed and accepted.

### Annotation format

While annotations can be represented in various formats, we used the BioC XML format due to several considerations. First, the format supports full-text articles and annotations representing both mention span (location) and entity identifier. Second, articles in the PMC text mining subset^25^ are already available in BioC XML format, and both the annotation interface, and the NER tool TaggerOne already support the format. Third, the format is simple and easy to modify, allowing additional analysis tools to be applied rapidly as needed.

Finally, TeamTat stores all annotations in BioC (XML and JSON), such that each annotation is specified by the character location on the document, the text mention, and its MeSH Identifier, and can be downloaded at any given time.

### The chemical entity recognition tool

As an initial demonstration of the value of NLM-Chem, we created an improved benchmark tool for chemical entity recognition and normalization. Recent deep learning methods, especially contextualized language models such as BERT^26^, have resulted in significant improvements in many natural language processing tasks, including named entity recognition. We therefore base the new tool on the bluebert (https://github.com/ncbi-nlp/bluebert) variant of BERT^18^, which was trained on PubMed abstracts and clinical notes from MIMIC-III (model BlueBERT-Base, Uncased, PubMed + MIMIC-III). The bluebert model was then fine-tuned on either the BC5CDR chemical named entity recognition corpus or a combination of BC5CDR and the NLM-Chem training set to provide chemical mention annotations.

To assign MeSH identifiers to the chemical mentions found by the bluebert NER tool, we paired it with a new normalization system which we have termed MTCR: Multiple Terminology Candidate Resolution. MTCR combines several methods, most well-known, into a pipeline optimized for chemical mention normalization, as follows:

1. MTCR attempts to assign MeSH identifiers to all spans recognized as chemicals by bluebert that contain at least one alphabetic character.
2. All abbreviations that appear in the mention text are resolved using abbreviation definitions identified by Ab3P^27^ in the full article text. Full-text articles may define abbreviations more than once; any definitions in conflict are resolved by selecting the definition that appears more frequently in PubMed/PMC.
3. The mention text is mapped to a set of candidate MeSH concepts using multiple string-matching methods. These methods are applied in sequence, with the first method that returns a non-zero number of MeSH concepts used as the overall result. The sequence is designed so earlier methods provide higher precision while later methods provide higher recall.
  - Exact match to MeSH: an exact match between mention text and MeSH concept names
  - Relaxed match to MeSH: The relaxed match method maps upper case text to lower case, maps non-ASCII characters to approximate ASCII equivalents, converts non-alphanumeric characters to spaces, then removes all spaces except those between two digits.
  - Relaxed plural match to MeSH. This method uses the same mapping as the relaxed match, but also processes each token using a conservative plural stemmer^28^.
  - Relaxed match to multiple chemical terminologies. This method uses the same string mapping as the relaxed match to MeSH, but applies it to all concept names from the FDA UNII database (https://fdasis.nlm.nih.gov/srs/), ChEBI^29^, the EPA DSSTox database^30^, and the UMLS^31^. Any matching concepts are then mapped to MeSH using a cross-reference directly to a MeSH identifier or an indirect cross-reference through a CAS, INCHI, INCHIKEY, UNII, SMILES, or EINECS identifier.
  - Relaxed plural match to multiple chemical terminologies. This method uses the same string mapping as the relaxed plural match to MeSH, and the same concept mapping as the relaxed match to multiple chemical terminologies.
4. The candidate MeSH identifiers for each mention are then resolved as follows:
  - Mentions that could not be mapped to any MeSH identifier are marked unknown.
  - Mentions are disambiguated by intersecting the set of candidate MeSH identifiers for the mention with the set of MeSH identifiers that appear in the document unambiguously (only one candidate), if present.

MeSH identifiers that do not appear in one of the MeSH hierarchies for chemicals are removed.

## Data Records

The NLM Chemical corpus is the largest corpus of full-text articles annotated with chemical entities at a high degree of granularity. The NLM Chemical corpus contains a total of 38,342 manual chemical mention annotations; corresponding to 4,867 unique chemical name annotations, normalized to 2,064 MeSH identifiers, extracted from 150 exhaustively examined full-text articles. Out of all mentions, 50% occur only once, 83% occur only in one article, 3.3% were unable to be normalized to a MeSH ID, and 11.3% were assigned to a combination of MeSH identifiers. Of the 2,064 unique MeSH assignments, 70% occur only in one article, and 15% contained two or more MeSH identifiers.

**Fig. 2:**
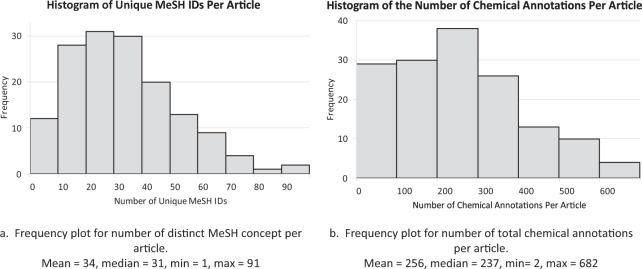
Frequency distribution of distinct MeSH annotations (**a**), and annotated chemical mentions (**b**) per article in the NLM-Chem corpus.

**Fig. 3:**
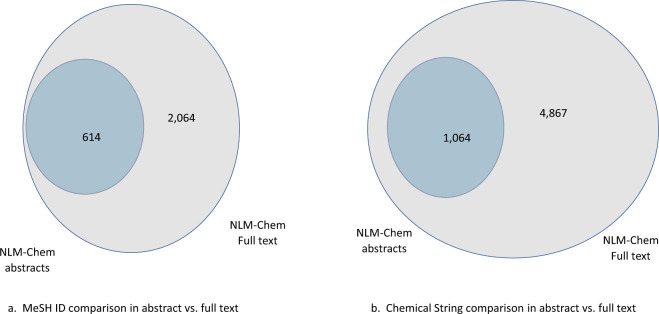
The distribution of chemical identifiers (**a**) and chemical mention annotations (**b**) in the NLM-Chem corpus abstracts vs the full-text articles.

Figure 2 depicts the distribution of unique MeSH identifiers and total chemical name annotations in each PMC article. Figure 3 shows the impact of annotations in the full text. As seen, the full text contains many more chemical annotations and a larger variety of both mentions as well as identifiers.

The NLM-Chem corpus is freely available from Dryad^19^ and at https://www.ncbi.nlm.nih.gov/research/bionlp/Data/, along with its annotation guidelines document.

An important step in corpus development is identifying a good split between the set of articles to be designated as the train set, development set and test set. An optimal split is useful for the development of new algorithms, meaning that the data needs to be similar and not have unexpected biases. For the NLM-Chem corpus, we prepared a split of 80/20/50 articles.

To ensure a similar distribution of articles in all the sets, we sampled proportionally from each annotation batch, selecting the articles so that the word distribution approximated the word distribution of the full corpus. This step ensured that we did not inadvertently split the dataset into defined clusters. Table 2 shows the distribution of annotations in each partition. Notice the high p-value, reflecting that the partitions are not different from each other.

**Table 2:** Distribution of chemical annotations for train/dev/test set.

Set	Number of Articles	Number of Chemical Annotations	Mean Number of Chemical Annotations Per Article	Stdev Of Chemical Annotations Per Article	P-Value Of T-Test Hypothesis That Sample Mean Is Significantly Different from Mean of Full Corpus
Train	80	22512	281.40	165.06	0.60
Dev	20	5546	277.30	167.46	0.85
Test	50	12409	248.18	157.40	0.41

## Technical Validation

### Corpus annotation quality (Inter-annotator agreement)

**Fig. 4:**
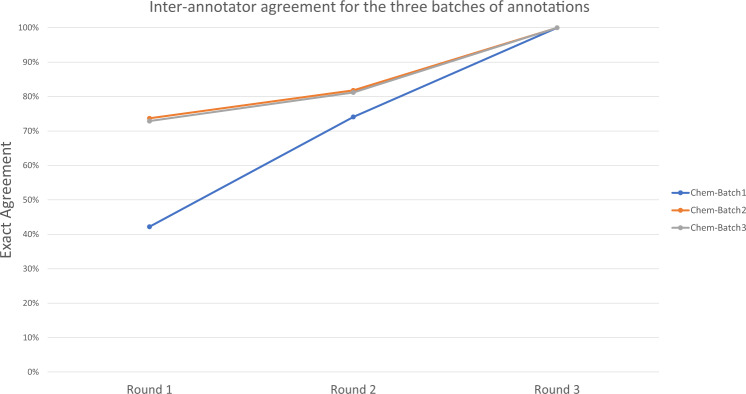
NLM-Chem corpus annotation quality. We have arranged the data to show the progression between annotation batches, as well as annotation rounds. The figure shows exact agreement measures and indicates that the two annotator partners agreed both on mention, and ID annotation. Annotation batch 1 is the time that the annotators were getting used to the task and writing the annotation guidelines. The numbers for batches 2 and 3 almost overlap with each-other, indicating the level of human agreement.

In order to make sure we are producing a quality resource, the inter-annotator agreement was checked after each annotation round. These results are shown in Table 3, and Fig. 4. In Fig. 4, we have arranged the data to show the progression between annotation batches, as well as annotation rounds. Exact agreement indicates that the two annotator partners agreed both on mention and ID annotation. The Mismatch ID case counts only the cases where annotators agree on the chemical mention, but they have selected different MeSH identifiers. The single-annotation case counts all mentions for which only one of the annotators supplied an annotation. If two annotations overlap but do not match exactly, they are counted as two single annotations. Please note that this is a very strict definition of annotation agreement.

**Table 3:** Inter-annotator agreement after annotation round 1, and round 2 for each annotation batch.

	Annotation Round 1			Annotation Round 2		
	Batch 1	Batch 2	Batch 3	Batch 1	Batch 2	Batch 3
Exact Agreement	42.20%	73.70%	72.90%	74.1%	81.8%	81.2%
Mismatch ID	8.60%	6.50%	9.80%	5.6%	1.0%	4.3%
Single - annotation	49.20%	19.80%	17.30%	20.3%	17.2%	14.5%

Similar to previous studies, analyzing the annotator disagreements that occurred in round 1 demonstrated that the conflicts were primarily due to missed annotations – that is, the span was annotated by one annotator but not the other – rather than differences in the mention boundary or the associated entity identifier.

The lowest annotation agreement is seen for annotation batch 1, round 1. This was the time when the annotators were getting used to the task and prepare the annotation guidelines. From round 1 to round 2 we see a significant increase in annotation agreement numbers. The inter-annotator agreement numbers in batches 2 and 3 are very similar, signifying that the true inter-annotator agreement of the task in general. Annotation round 3 involves pair-wise meetings between the annotators, during which they discuss and reconcile their differences to produce 100% agreement. Note that annotator partners are only revealed during round 3. Averaged load distribution amongst annotator pairs and random anonymous pairings between annotators are used to control annotation bias.

Another significant measure of corpus quality is the level of agreement of the final annotations of the corpus with the proposed tool annotations that the annotators are given in the beginning. Again, we show each annotation batch separately because we used the finalized annotations after each batch to retrain and improve the model recognition power before we supplied pre-annotations for the next batch. Annotators were encouraged to review the tool results critically, and the numbers shown in Table 4, reflect the fact that the tool did not bias the annotators when making their decisions. It is important to see that the annotators trust the tool more on finding a chemical mention than on finding the correct MeSH identifier.

**Table 4:** Accuracy of pre-annotations.

	Chemical mention recognition			Chemical ID recognition		
	Batch 1	Batch 2	Batch 3	Batch 1	Batch 2	Batch 3
Precision	68.6%	83.0%	73.8%	49.7%	68.1%	74.0%
Recall	54.6%	68.0%	71.6%	57.6%	67.9%	75.8%
F-measure	60.8%	74.8%	72.7%	53.3%	68.0%	74.9%

### The NLM-Chem corpus characteristics

**Fig. 5:**
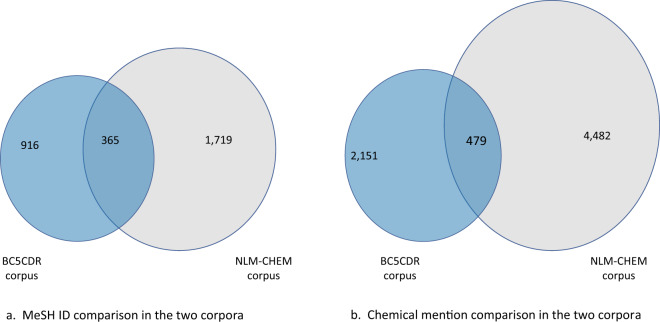
A comparison of Chemical mention (**a**) and ID (**b**) composition of the BC5CDR and NLM-CHEM corpora. We show the chemical mentions and identifiers that they differ, and the ones that they have in common.

The NLM-Chem corpus is a rich corpus created for chemical named entity recognition. We compared the NLM-Chem corpus with both the BC5CDR and ChemDNER corpora. Figure 5 shows a comparison with BC5CDR, because we can compare this both on the mention and ID level. The BC5CDR corpus contains PubMed abstracts annotated with chemical and disease mentions and chemical-disease relations. It was introduced as part of a shared task at BioCreative 5 and is annotated with mention spans and MeSH ID concept identifiers. It can therefore be used to train both named entity recognition and normalization systems. In addition, we compared the Chemical mentions in NLM-Chem with ChemDNER. Our analysis shows that 70% of the (unique) Chemical names annotated in the NLM-Chem corpus are new mentions, not previously identified in the previous corpora.

As shown in Fig. 5, annotating full text significantly increases both diversity and synonymy in the NLM-Chem corpus. More than 70% of the unique MeSH assignments, and more than 78% of unique chemical names come from the full text. Such diversity is important for PubMed search. As expected, analyzing the chemical mention annotations assigned to the same MeSH ID showed the differences to be lexical variations, abbreviated forms, alternative spellings, meronyms and synonyms.

Table 5 shows the distribution of chemical annotations over the different sections of a full-text article. The data varied within the full text sections and we were not able to identify any certain category of chemicals prevalent to a specific section. While the general expectation of the MeSH indexers was that most of the chemicals mentioned in the Methods section would be reagents, a detailed inspection of the unique chemicals listed in the Methods section also revealed many occurrences of specific chemicals. After this analysis we can conclude that for many search and indexing tasks, processing the whole article is useful.

**Table 5:** Distribution of Chemical annotations in full text sections.

	Chemical mentions			Chemical MeSH IDs	
	Total	Unique	Unique to Section	Unique	Unique to Section
Title	169	164	40	149	1
Abstract	2,421	989	119	611	6
Intro	4,236	1,461	543	812	186
Methods	7,696	2,228	1,098	1,194	368
Results	11,910	1,957	644	1,124	191
Discussion	5,670	1,048	282	706	131
Table and figure captions	5,480	1,220	208	743	22

We also analyzed the journals which published the 150 articles. There were 61 different journals, the most common are listed in Table 6. Eight of the ten most common journals are related to chemistry, while the remaining two (PLoS One and BioMed research international) are broad topic.

**Table 6:** Top ten journals in the NLM-Chem corpus.

NUMBER OF ARTICLES	JOURNAL NAME
26	International journal of molecular sciences
20	PLoS One
10	The Journal of biological chemistry
6	Beilstein journal of organic chemistry
6	Molecular vision
5	Molecular pain
4	BioMed research international
4	Chemical science
4	Molecular cancer
3	Acta crystallographica. Section E, Structure reports online

We noted that 37% of the articles in NLM Chem were published in just three journals. To determine whether our selection process skewed the journal distribution, we analyzed the nearly 300,000 full-text articles that pass our filtering steps, including the requirement for CC-BY or CC-0 licensing. We found that the journal distribution of the full filtered set is also highly skewed, with over 30% of the articles appearing in PLoS One (data not shown). Moreover, we found that a sample of 150 articles chosen randomly would likely contain at least 15 more articles from PLoS One, while the journals related to chemistry would each be expected to appear slightly less frequently (hypergeometric distribution, 95% confidence interval). We thus conclude that our selection process demonstrates a preference for articles enriched in chemical content while reflecting the diversity of the literature overall, as designed.

## Usage Notes

### Chemical named entity recognition evaluation

As a preliminary demonstration of the value of a full-text corpus for chemical named entity recognition and normalization, we prepared an experiment to show the improvement in performance on annotating chemicals in full-text articles when chemical annotations from full-text articles are available for training. For named entity recognition, each mention span was considered separately. A prediction only counted as a true positive if both the start and end characters matched (strict boundaries). For normalization, the evaluation compared the set of concepts annotated in the full-text document and the set of concepts predicted as present. In both cases, precision was defined as the number of true positives divided by the number of predictions, recall as the number of true positives divided by the number of annotations, and F-score as the harmonic mean of precision and recall.

This experiment consisted of four experimental conditions. These conditions compare the difference in the performance of two different models on the NLM-Chem test set when trained on the BC5CDR training data, which contains only titles and abstracts, and the same model when trained with the NLM-Chem training set. Each condition is evaluated on the NLM-Chem test dataset.

The first condition, shown in Table 7, first row, consisted of training the TaggerOne system for joint named entity recognition and normalization on the chemicals in the BC5CDR corpus (training and development sets), using the BC5CDR sample set as a development set. Training was stopped when performance on the development set did not improve after five iterations through the training data.

**Table 7:** Initial text mining evaluation, showing performance results on the NLM-Chem test set, when the training is performed on the listed combinations of training sets, and controlled on the development set.

System	Training Corpus	Chemical Mention Recognition			MeSH ID Recognition		
		Precision	Recall	F-measure	Precision	Recall	F-measure
TaggerOne	BC5CDR	0.580	0.414	0.483	0.470	0.508	0.488
	BC5CDR + NLM-CHEM	0.724	0.615	0.665	0.653	0.681	0.666
Bluebert + MTCR	BC5CDR	0.731	0.523	0.610	0.792	0.599	0.682
	BC5CDR + NLM-CHEM	**0.810**	**0.711**	**0.757**	**0.822**	**0.728**	**0.772**

For the second condition, we trained the TaggerOne model using a combination of the BC5CDR corpus (training and development sets) and the NLM-Chem train dataset as the training data, with a combination of the BC5CDR sample set and NLM-Chem development set as the development set. Training was stopped when performance on the development set did not improve after five iterations through the training data. This evaluation clearly shows a sharp jump in performance obtained when trained using a combination of PubMed abstracts and full-text documents.

For the third condition, we trained the bluebert deep learning model^18^ on the BC5CDR training and development sets. We see a great improvement in the precision and recall of chemical names, due to the deep learning architecture’s ability to use the context and generalize from it. The normalization results use the bluebert output to locate the chemical mentions, then apply the MTCR method. Again, we see an improvement in the accuracy of correctly identifying the MeSH identifiers.

For the fourth experimental condition, we trained the bluebert deep learning model using a combination of the BC5CDR corpus (training and development sets) and the NLM-Chem training set (similar to the second condition above), again using the MTCR method for MeSH ID recognition. Compared with the bluebert results using BC5CDR for training, we see a great improvement in both precision and recall for both mentions and MeSH IDs, confirming the significant importance of annotated full-text corpora for training and evaluating the performance of models on full-text articles. Note that these results are not far below the human inter-annotator agreement values.

NLM-Chem is a high-quality corpus, doubly annotated by ten NLM indexers, in three rounds of annotation, and all annotator disagreements have been resolved. NLM-Chem corpus is currently the largest corpus of full-text articles annotated with chemical entities at a high degree of granularity. It contains a total of 38,342 manual chemical mention annotations; corresponding to 4,867 unique chemical name annotations, normalized to 2,064 MeSH identifiers, extracted from 150 exhaustively examined full-text articles. The articles were carefully selected from the PMC Open Access dataset and cover 61 journals. The data is split in a train/development/test set arrangement that approximates a fair word distribution. The NLM-Chem corpus will be invaluable for advancing text-mining techniques for chemical extraction tasks in biomedical text.

We created a benchmark chemical entity recognition and normalization system to provide a robust method that could translate to real-life applications. Our system uses deep learning to recognize chemical mentions and a multi-terminology candidate resolution (MTCR) architecture to map each mention to a MeSH identifier. MTCR uses multiple string matching methods and chemical terminologies to generate candidate mappings, resolves ambiguous mappings, and eliminates mappings that are unlikely to be chemicals. Our system results in significantly higher performance than available with our previous chemical recognition tool and can identify chemicals in the NLM-Chem test dataset with performance close to the human inter-annotator agreement.

Because our goal is to provide practical benefits with our NLP research, NLM-Chem is available from Dryad^19^ and at https://www.ncbi.nlm.nih.gov/research/bionlp/Data/ and the chemical recognition results have been streamlined to process all PubMed articles in daily updates: https://www.ncbi.nlm.nih.gov/research/bionlp/APIs/ and from https://www.ncbi.nlm.nih.gov/research/pubtator/.
